# Determinants of complementary and integrative medicine use in inflammatory bowel disease: a focus on probiotics, prebiotics, and fermentable dietary fibres

**DOI:** 10.3389/fmed.2025.1641767

**Published:** 2025-10-09

**Authors:** Ammar H. Keshteli, Richa Chibbar, Melissa Silva, Karen Danois, Rosica Valcheva, Eytan Wine, Levinus A. Dieleman

**Affiliations:** ^1^Division of Gastroenterology, Department of Medicine, University of Alberta, Edmonton, AB, Canada; ^2^Division of Pediatric Gastroenterology, Department of Pediatrics, University of Alberta, Edmonton, AB, Canada

**Keywords:** inflammatory bowel disease, complementary and integrative medicine, complementary and alternative medicine, prebiotics, probiotics, dietary fibres

## Abstract

**Background:**

Complementary and integrative medicine (CIM) modalities, particularly probiotics, prebiotics, and fermentable dietary fibers (PPF) use in IBD patients is common and increasing, particularly for symptom management. This study aimed to assess the prevalence of CIM and PPF use among IBD patients and to identify potential demographic and clinical factors associated with utilization.

**Methods:**

This was a cross-sectional study of adult IBD patients at a tertiary IBD centre in Western Canada. A self-administered questionnaire and chart review were performed, focusing on demographic and clinical characteristics, CIM and PPF use in the past year (current) and/or lifetime, and sources of awareness about PPF products.

**Results:**

A total of 267 patients were included, 182 with CD and 85 with UC. Overall, 89.9% of participants reported CIM use in the current year, while the current and lifetime prevalence of PPF use was 51 and 63%, respectively. UC diagnosis was associated with increased likelihood of current PPF use (OR: 1.91, 95% CI: 1.10–3.12). Holding a university degree was associated with increased likelihood of lifetime PPF use (OR: 2.21, 95% CI: 1.07–4.55). PPF awareness through gastroenterologists (OR: 3.19, 95% CI: 1.55–6.58) was significantly associated with lifetime PPF use.

**Conclusion:**

Use of CIM modalities such as PPF is common among IBD patients. This study found that lifetime PPF use was associated with higher level of education and awareness through gastroenterologists. Healthcare providers, and specifically gastroenterology specialists, should routinely inquire about PPF use and educate IBD patients. Further studies are required to determine the benefit derived from these products.

## Introduction

Inflammatory bowel disease (IBD) is a chronic, progressive, relapsing-remitting inflammation of the gastrointestinal tract, consisting primarily of Crohn’s disease (CD) and ulcerative colitis (UC). It is thought to be resulted from a complex interaction characterized by an abnormal immune response to altered gut microbiota in genetically susceptible individuals (1, 2). The goal of therapy is to induce and maintain a steroid-free deep remission, typically achieved with conventional medical therapies directed against acute and chronic intestinal inflammation and sometimes requiring surgical management (1, 2). Though mostly efficacious, pharmaceutical therapies are costly and can be limited by potential adverse effects prompting many patients to seek other treatments alongside conventional care (3).

According to the National Center for Complementary and Integrative Health (NCCIH), CAM is defined as a diverse group of medical and healthcare systems, practices, and products that are not generally considered part of conventional medicine. CAM approaches can be categorized based on their primary therapeutic input, including nutritional (e.g., special diets, dietary supplements, herbs, probiotics), psychological (e.g., mindfulness), physical (e.g., massage, spinal manipulation), and combined approaches (e.g., yoga, acupuncture, dance, mindful eating) (4). In conditions such as IBD, these non-mainstream approaches are used alongside conventional medical treatments rather than as replacements and are therefore not truly “alternative.” Following NCCIH recommendations, these approaches fall under integrative health, which combines conventional and complementary interventions in a coordinated, multimodal manner, with an emphasis on whole-person care rather than targeting individual organ systems. Accordingly, the preferred replacement for the term CAM in an IBD context is complementary and integrative medicine (CIM).

CIM use is common among adults with IBD, however supporting evidence of their effectiveness in IBD remains limited and conflicting (5–8). Probiotics, prebiotics, and fermentable dietary fibres (PPF) are amongst the most commonly forms of CIM used by IBD patients (8, 9). The rationale behind their use lies in the potential role of gut microbiota in the pathophysiology of IBD. Altered gut microbial composition and function, along with other contributing factors such as genetics, immune dysregulation, and environmental influences, play a major role in the development and perpetuation of the disease (10–12). Dysbiosis, an imbalance of the gut microbiota, associated with both endogenous (e.g., immune system interactions, epithelial cell responses) and exogenous factors (e.g., medications, surgery, diet), ultimately contributes to chronic intestinal inflammation and damage (10, 13). Therefore, it has been suggested that modulating the gut microbiota through approaches such as fecal microbial transplantation, dietary interventions, and PPF consumption may benefit IBD patients by restoring the dynamic balance between the gut microbiota and host mucosal immune system mechanisms (14). While clinical trials on the effectiveness of PPFs in IBD remain inconclusive (15, 16), a growing number of patients use them for different reasons such as the prevention of disease relapse, symptom control, and improvement of general health.

With growing use of CIM modalities such as PPFs, it is necessary to examine the potential factors that influence their utilization through well-designed epidemiological studies. Therefore, we conducted the present study to estimate the proportion of our IBD patients utilizing PPF, and to determine the demographic and clinical factors associated with use among our IBD patient population.

## Methods

This cross-sectional study was conducted at the University of Alberta Gastroenterology outpatient IBD clinic and an infusion clinic in Edmonton, Alberta, Canada. Eligible participants were 18 years or older with an established IBD diagnosis, confirmed by a physician. Those unable to speak or write fluently in English were excluded. The estimated number of patients attending the IBD clinic was ≈2,800. A sample size of 287 was calculated to estimate the prevalence of PPF use [assumed 58% based on the proportion of probiotic and prebiotic use reported by Hedin et al. (17)] with a 5% margin of error and 93% confidence level, applying a population size adjustment.

All participants provided informed consent before completing the questionnaire. Care providers were not present during the data collection process. Responses were anonymized and kept confidential, with no identifying information recorded. The study protocol was approved by the University of Alberta Human Research Ethics Board (No. Pro00064575).

Participants were asked to complete a self-administered questionnaire in the clinic waiting room, either before or after their clinic visit. The questionnaire included twenty items covering demographic information (gender, age, ethnicity, and education level), summarized clinical details (disease diagnosis, duration, medications, and relapse history), and CIM usage in the past year (e.g., vitamins, minerals, herbal supplements, massage, chiropractic care, yoga, meditation, and acupuncture). To assess PPF use, participants were provided with a comprehensive list of PPF products available in Canada and asked to indicate if they had history of using any of the products (lifetime PPF use) or had used them within the past year (current PPF use). They were also asked to identify their sources of knowledge about PPF use (e.g., social media, family and friends, internet, advertisements, family physician, gastroenterologist, dietitian or nurse, and pharmacist). A trained interviewer was present during data collection to address participant questions and ensure the questionnaires were completed accurately and thoroughly.

Following the clinic visit, a detailed chart review was conducted to validate the accuracy of the data collected via the self-administered questionnaire and to gather additional clinical information. This included surgical history, current medications, past corticosteroid use, extraintestinal manifestations of IBD, current partial Mayo score for UC, Harvey-Bradshaw Index for CD, Montreal classification, disease flare frequency over the past 2 years, and average serum C-reactive protein (CRP) levels over the past year. Active disease in UC was defined as a current partial Mayo score between 2 and 9 (18), while in CD, it was defined as a Harvey-Bradshaw Index ≥ 5 (19). Elevated CRP was defined as serum CRP > 8 mg/L (20). For the purposes of this study, flares were defined as a combination of clinical symptoms and endoscopic and/or histological scores. Clinical symptoms pertaining to a flare included diarrhea ≥ 3 days, obvious blood in the stool, bloating and/or abdominal pain. Symptoms such as presence of fever > 38° C, loss of appetite, nausea/vomiting, fatigue and change in health status were also considered.

Quantitative variables are presented as mean ± standard deviation (SD). Normally distributed data were compared between groups using independent sample t-tests, while non-normally distributed data (assessed by the Kolmogorov-Smirnov test) were analyzed using the Mann-Whitney U test. Qualitative variables are expressed as frequencies and percentages and were compared using Chi-square or Fisher’s exact tests, as appropriate. Binary logistic regression was performed to identify predictors of CIM or PPF use. All statistical analyses were conducted using IBM SPSS Statistics software (version 26.0, Armonk, NY, USA: IBM Corp). A two-tailed *p*-value of < 0.05 was considered statistically significant.

## Results

### Demographics and clinical phenotypes

Of the 280 patients invited to participate, 13 were excluded from analysis: 4 lacked a confirmed IBD diagnosis, and 9 had IBD-unclassified. Among the remaining 267 individuals with confirmed diagnosis of IBD, 182 (68.2%) were diagnosed with CD and 85 (31.8%) with UC. The mean age was 42.6 ± 15.7 years and 53.9% were female. Demographic and clinical characteristics of participants and comparisons based on IBD subtype (UC vs. CD), are presented in Table 1. Patients with UC were significantly younger, had shorter disease duration, were less likely to be Caucasian, and more likely to be on 5-ASA therapy versus patients with CD. In contrast, those with CD were more likely to be on biologic therapy, have a history of IBD-related surgery, and to exhibit extraintestinal manifestations of IBD.

**Table 1:** Comparison of demographic and clinical characteristics between ulcerative colitis (UC) and Crohn’s disease (CD) patients.

	Total (*n* = 267)	Ulcerative colitis (*n* = 85)	Crohn’s disease (*n* = 182)	*p*-value
Age, yr	42.6 ± 15.7	39.6 ± 15.2	44.0 ± 15.8	0.04
Age at diagnosis, yr	29.3 ± 14.0	30.2 ± 13.6	28.8 ± 14.2	0.46
Disease duration, yr	13.3 ± 11.2	9.4 ± 9.2	15.1 ± 11.6	**<0.001**
Females, *n* (%)	144 (53.9)	45 (52.9)	99 (54.4)	0.82
Education, *n* (%)				
Less than high school	76 (28.5)	19 (22.4)	57 (31.3)	
High school or college	119 (44.6)	37 (43.5)	82 (45.1)	0.13
University degree	72 (27.0)	29 (34.1)	43 (23.6)	
Caucasian, *n* (%)	244 (91.4)	72 (84.7)	172 (94.5)	0.01
UC Montreal classification, *n* (%)				
Proctitis		5 (5.9)		
Left-sided colitis		18 (21.2)		
Pancolitis		62 (72.9)		
CD Montreal classification, *n* (%)				
Ileal			47 (25.8)	
Colonic			34 (18.7)	
Ileocolonic			101 (55.5)	
Upper gastrointestinal			38 (20.9)	
Inflammatory			72 (39.6)	
Stricturing			92 (50.5)	
Penetrating			42 (23.1)	
Stricturing and penetrating			15 (8.2)	
Perianal			61 (33.5)	
IBD medication, *n* (%)				
No medication	35 (13.1)	10 (11.8)	25 (13.7)	0.66
5-aminosalicylic acid	85 (31.8)	55 (64.7)	30 (16.5)	**<0.001**
Immunomodulators	94 (35.2)	23 (27.1)	71 (39.0)	**0.06**
Biologics	157 (58.8)	34 (40.0)	123 (67.6)	**<0.001**
Current corticosteroids	10 (3.7)	4 (4.7)	6 (3.3)	0.57
Past corticosteroids	215 (80.9)	69 (81.2)	146 (80.2)	0.85
Surgical history, *n* (%)	79 (29.6)	2 (2.4)	77 (42.3)	**<0.001**
Extraintestinal manifestation, *n* (%)	133 (49.8)	15 (17.6)	118 (64.8)	**<0.001**
Number of flares in the last 2 yr	1.4 ± 1.2	1.7 ± 1.3	1.2 ± 1.2	**0.01**
Harvey Bradshaw Index			1.4 ± 0.7	
Partial Mayo score		1.5 ± 0.8		
Active disease, *n* (%)	80 (30.0)	32 (37.6)	48 (26.4)	0.06
CRP > 8 mg/L in the past year, *n* (%)	81 (33.6)	34 (43.0)	70 (40.7)	0.73

IBD, inflammatory bowel disease; CRP, C-reactive protein.

Bold values indicate statistically significant *p*-values (*p* < 0.05).

### CIM use

**Figure 1:**
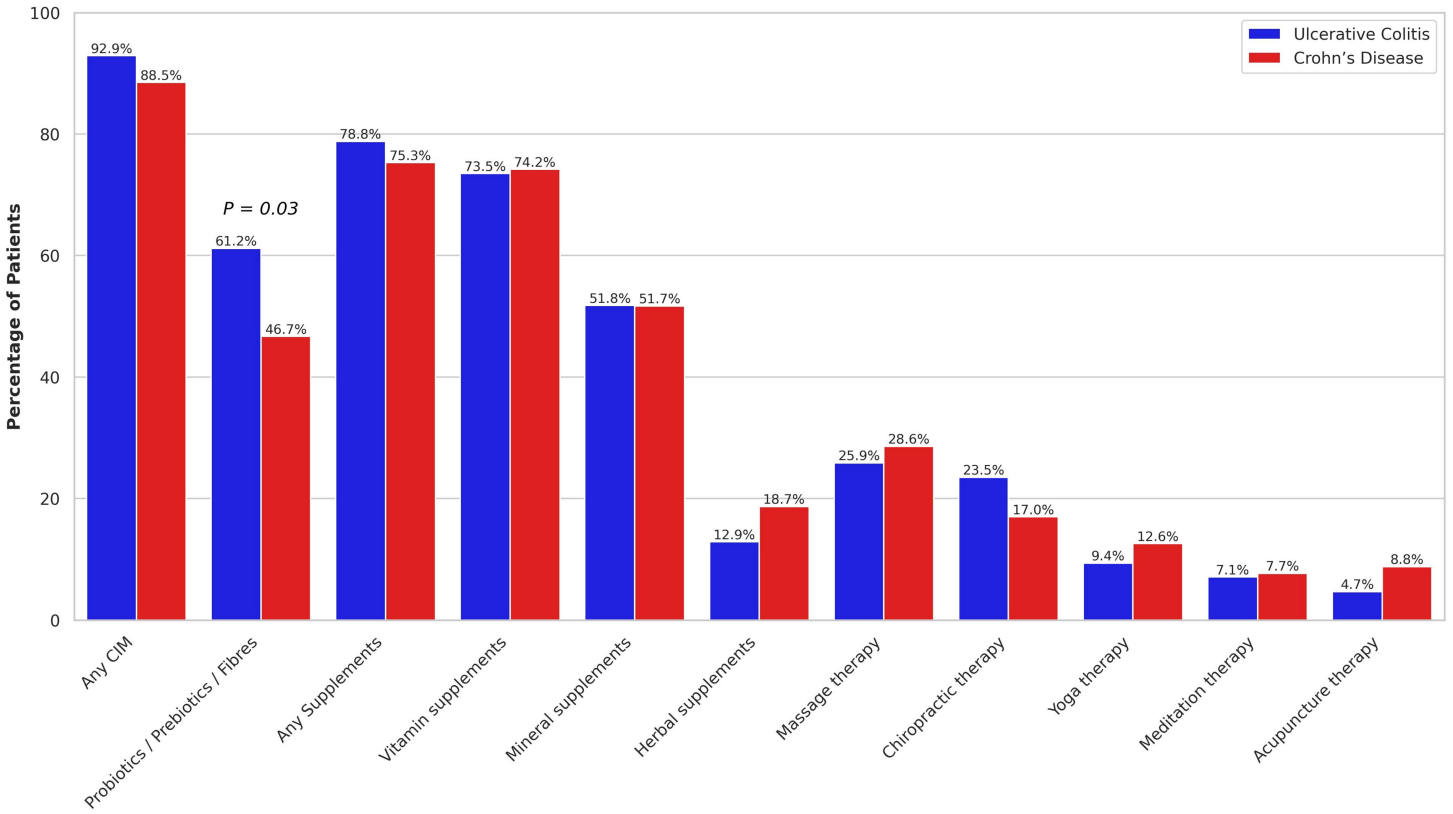
Comparison of complementary and integrative medicine (CIM) use in the past year between ulcerative colitis and Crohn’s disease patients. Only *p*-values < 0.05 are shown.

Overall, 89.9% of participants reported using any CIM modalities within the past year. The most common CIM modalities included supplements (76.4%), PPFs (51.3%), and massage therapy (27.7%). Comparison of CIM and its modalities use between UC and CD patients is presented in Figure 1. PPF use was significantly higher in UC (61.2%) compared to CD patients (46.7%) (OR: 1.80, 95% CI: 1.06–3.04). The use of other CIM modalities was comparable between UC and CD patients.

The relationship between demographic and clinical characteristics and CIM use is presented in Table 2. None of the demographic or clinical factors showed a statistically significant association with CIM use neither in univariate nor in multivariable analysis.

**Table 2:** Association of complementary and integrative medicine use with demographic and clinical characteristics of inflammatory bowel disease patients.

	Complementary and integrative medicine use		*p*-value
	Yes (*n* = 240)	No (*n* = 27)	
Age, yr	43.0 ± 15.8	39.0 ± 14.8	0.21
Females, *n* (%)	134 (55.8)	10 (37.0)	0.06
Caucasian, *n* (%)	220 (91.7)	24 (88.9)	0.63
Education, *n* (%)			
Less than high school	67 (27.9)	9 (33.3)	
High school or college	106 (44.2)	13 (48.1)	0.57
University degree	67 (27.9)	5 (18.5)	
Ulcerative colitis (UC), *n* (%)	79 (32.9)	6 (22.2)	0.26
Crohn’s disease (CD), *n* (%)	161 (67.1)	21 (77.8)	
Disease duration, yr	13.6 ± 11.3	10.9 ± 10.4	0.24
UC Montreal classification, *n* (%)			
Proctitis	5 (6.3)	0 (0.0)	0.76
Lef-sided colitis	17 (21.5)	1 (16.7)	
Pancolitis	57 (72.2)	5 (83.3)	
CD Montreal classification, *n* (%)			
Ileal	42 (26.1)	5 (23.8)	0.82
Colonic	31 (19.3)	3 (14.3)	0.58
Ileocolonic	88 (54.7)	13 (61.9)	0.63
Upper gastrointestinal	34 (21.1)	4 (19.0)	0.83
Inflammatory	67 (41.6)	5 (23.8)	0.12
Stricturing	81 (50.3)	11 (52.4)	0.86
Penetrating	35 (21.7)	7 (33.3)	0.24
Stricturing and penetrating	14 (8.7)	1 (4.8)	0.54
Perianal	53 (32.9)	8 (38.1)	0.64
IBD medication, *n* (%)			
No medication	32 (13.3)	3 (11.1)	>0.99
5-aminosalicylic acid	80 (33.3)	5 (18.5)	0.12
Immunomodulators	82 (34.2)	12 (44.4)	0.29
Biologics	140 (58.3)	17 (63.0)	0.64
Current corticosteroids	6 (3.2)	4 (5.0)	0.49
Past corticosteroids	151 (80.7)	64 (80.0)	0. 89
Surgical history, *n* (%)	68 (28.3)	11 (40.7)	0.18
Extraintestinal manifestation, n (%)	120 (50.0)	13 (48.1)	0.86
Number of flares in the last 2 yr	1.4 ± 1.3	1.3 ± 1.2	0.92
Active disease, *n* (%)	53 (28.3)	27 (33.8)	0.38
CRP > 8 mg/L in the past year, *n* (%)	68 (38.4)	36 (48.6)	0.13

IBD, inflammatory bowel disease; CRP, C-reactive protein.

### PPF use

Overall, 51% of participants reported current PPF use, and 63% reported lifetime PPF use. Among current PPF users, probiotics were the most commonly used (90.2%), followed by dietary fiber supplements (23.5%) and prebiotics (11.8%). Since PPF use was significantly higher in UC patients and given the differences in demographic and clinical characteristics between UC and CD patients (Table 1), further analyses to identify determinants of PPF use were conducted separately for each IBD subtype.

In univariate analysis, none of the demographic or clinical characteristics were associated with current or lifetime PPF use in UC (Table 3) or CD (Table 4) patients. However, in multivariable analysis, a diagnosis of UC was associated with increased odds of current PPF use (OR: 1.91, 95% CI: 1.10–3.12, *p* = 0.02). Furthermore, a university degree was associated with higher odds of lifetime PPF use among IBD patients (OR: 2.21, 95% CI: 1.07–4.55, *p* = 0.03).

**Table 3:** Demographic and clinical characteristics of ulcerative colitis patients according to their current and lifetime probiotics, prebiotics, and fermentable dietary fibres consumption status.

	Current consumption		*p*-value	Lifetime consumption		*p*-value
	Yes (*n* = 52)	No (*n* = 33)		Yes (*n* = 57)	No (*n* = 28)	
Age, yr	39.8 ± 15.3	39.5 ± 15.1	0.93	38.8 ± 15.1	41.4 ± 15.4	0.46
Disease duration, yr	9.5 ± 9.1	9.3 ± 9.3	0.92	9.3 ± 8.8	9.6 ± 10.0	0.89
Females, *n* (%)	30 (57.7)	15 (45.5)	0.27	33 (57.9)	12 (42.9)	0.19
Education, *n* (%)						
Less than high school	11 (21.2)	8 (24.2)	Ref	11 (19.3)	8 (28.6)	Ref
High school or college	22 (42.3)	15 (45.5)	0.91	23 (40.4)	14 (50.0)	0.76
University degree	19 (36.5)	10 (30.3)	0.59	23 (40.4)	6 (21.4)	0.12
University degree, *n* (%)	19 (36.5)	10 (30.3)	0.56	23 (40.4)	6 (21.4)	0.08
Caucasian, *n* (%)	45 (86.5)	27 (81.8)	0.56	49 (86.0)	23 (82.1)	0.65
Montreal classification, *n* (%)						
Proctitis	5 (9.6)	0 (0.0)	0.18	5 (8.8)	0 (0.0)	0.27
Lef-sided colitis	11 (21.2)	7 (21.2)		12 (21.1)	6 (21.4)	
Pancolitis	36 (69.2)	26 (78.8)		40 (70.2)	22 (78.6)	
IBD medication, *n* (%)						
No medication	6 (11.5)	4 (12.1)	0.28	8 (14.0)	2 (7.1)	0.35
5-aminosalicylic acid	32 (61.5)	23 (69.7)	0.44	35 (61.4)	20 (71.4)	0.36
Immunomodulators	16 (30.8)	7 (21.2)	0.84	17 (29.8)	6 (21.4)	0.41
Biologics	23 (44.2)	11 (33.3)	0.91	25 (43.9)	9 (32.1)	0.3
Current corticosteroids	2 (3.8)	2 (6.1)	0.64	2 (3.5)	2 (7.1)	0.46
Past corticosteroids	41 (78.8)	28 (84.8)	0.49	45 (78.9)	24 (85.7)	0.45
Surgical history, *n* (%)	1 (1.9)	1 (3.0)	0.74	2 (3.5)	0 (0.0)	>0.99
Extraintestinal manifestation, *n* (%)	10 (19.2)	5 (15.2)	0.3	11 (19.3)	4 (14.3)	0.57
Number of flares in the last 2 yr	1.6 ± 1.2	1.8 ± 1.4	0.58	1.5 ± 1.2	2.0 ± 1.5	0.13
Partial Mayo score	1.5 ± 0.8	1.6 ± 0.8	0.75	1.5 ± 0.8	1.6 ± 0.8	0.81
Active disease, *n* (%)	19 (36.5)	13 (39.4)	0.79	21 (36.8)	11 (39.3)	0.83
CRP > 8 mg/L in the year, n (%)	19 (38.8)	15 (50.0)	0.33	21 (38.9)	13 (52.0)	0.27

IBD, inflammatory bowel disease; CRP, C-reactive protein.

**Table 4:** Demographic and clinical characteristics of Crohn’s disease patients according to their current and lifetime probiotics, prebiotics, and fermentable dietary fibres consumption status.

	Current consumption		*p*-value	Lifetime consumption		*p*-value
	Yes (*n* = 85)	No (*n* = 97)		Yes (*n* = 111)	No (*n* = 71)	
Age, yr	44.3 ± 15.3	43.7 ± 16.3	0.78	44.7 ± 16.0	42.8 ± 15.7	0.41
Disease duration, yr	15.8 ± 12.4	14.5 ± 11.0	0.45	15.6 ± 12.1	14.3 ± 10.9	0.46
Females, *n* (%)	50 (58.8)	49 (50.5)	0.26	64 (57.7)	35 (49.3)	0.27
Education, *n* (%)						
Less than high school	26 (30.6)	31 (32.0)	Ref	30 (27.0)	27 (38.0)	Ref
High school or college	41 (48.2)	41 (42.3)	0.61	52 (46.8)	30 (42.3)	0.21
University degree	18 (21.2)	25 (25.8)	0.71	29 (26.1)	14 (19.7)	0.14
University degree	18 (21.2)	25 (25.8)	0.47	29 (26.1)	14 (19.7)	0.32
Caucasian, *n* (%)	80 (94.1)	92 (94.8)	0.83	104 (93.7)	68 (95.8)	0.55
Montreal classification, *n* (%)						
Colonic	18 (21.2)	16 (16.5)	0.42	22 (19.8)	12 (16.9)	0.62
Ileocolonic	46 (54.1)	55 (56.7)	0.73	59 (53.2)	42 (59.2)	0.43
Upper gastrointestinal	13 (15.3)	25 (25.8)	0.08	20 (18.0)	18 (25.4)	0.24
Inflammatory	33 (38.8)	39 (40.2)	0.85	43 (38.7)	29 (40.8)	0.78
Stricturing	44 (51.8)	48 (49.5)	0.76	59 (53.2)	33 (46.5)	0.38
Penetrating	20 (23.5)	22 (22.7)	0.89	23 (20.7)	19 (26.8)	0.35
Stricturing and penetrating	8 (9.4)	7 (7.2)	0.59	9 (8.1)	6 (8.5)	0.94
Perianal	28 (32.9)	33 (34.0)	0.88	36 (32.4)	25 (35.2)	0.7
IBD medication, *n* (%)						
No medication	9 (10.6)	16 (16.5)	0.25	16 (14.4)	9 (12.7)	0.74
5-aminosalicylic acid	18 (21.2)	12 (12.4)	0.11	19 (17.1)	11 (15.5)	0.77
Immunomodulators	33 (38.8)	38 (39.2)	0.96	39 (35.1)	32 (45.1)	0.18
Biologics	58 (68.2)	65 (67.0)	0.86	75 (67.6)	48 (67.6)	>0.99
Current corticosteroids	4 (4.7)	2 (2.1)	0.32	5 (4.5)	1 (1.4)	0.41
Past corticosteroids	72 (84.7)	74 (76.3)	0.16	92 (82.9)	54 (76.1)	0.26
Surgical history, *n* (%)	38 (44.7)	39 (40.2)	0.54	52 (46.8)	25 (35.2)	0.12
Extraintestinal manifestation, *n* (%)	54 (63.5)	64 (66.0)	0.73	72 (64.9)	46 (64.8)	0.99
Number of flares in the last 2 yr	1.3 ± 1.3	1.2 ± 1.2	0.73	1.2 ± 1.2	1.2 ± 1.2	0.78
Harvey Bradshaw Index	1.4 ± 0.7	1.4 ± 0.7	0.73	1.4 ± 0.7	1.4 ± 0.8	0.44
Active disease, *n* (%)	21 (24.7)	27 (27.8)	0.63	28 (25.2)	20 (28.2)	0.66
CRP > 8 mg/L in the past year, *n* (%)	33 (41.3)	37 (40.2)	0.89	40 (38.1)	30 (44.8)	0.38

IBD, inflammatory bowel disease; CRP, C-reactive protein.

### Sources of PPF awareness

Overall, 243 (91.0%) patients reported general awareness of PPFs. General awareness was significantly associated with female gender (OR: 2.54, 95% CI: 1.05–6.16). No other demographic or clinical characteristics were associated with general awareness of PPFs. General awareness of PPFs was significantly associated with both current (OR: 6.05, 95% CI: 2.00–18.21) and lifetime (OR: 7.74, 95% CI: 2.79–21.49) PPF use.

**Figure 2:**
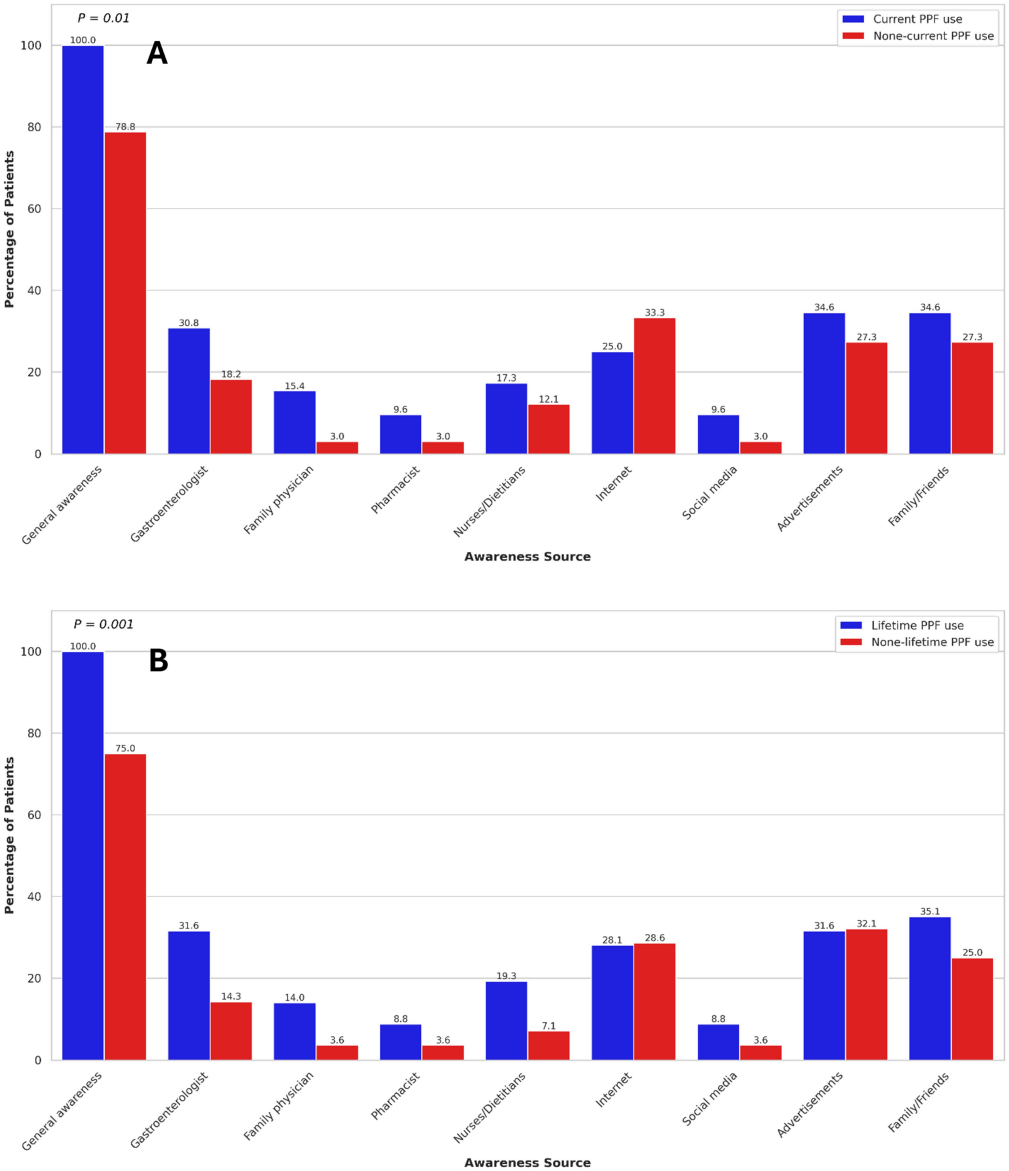
Association of current **(A)** and lifetime **(B)** probiotics, prebiotics, and fermentable dietary fibres use with sources of awareness in ulcerative colitis patients. Only *p*-values < 0.05 are shown.

**Figure 3:**
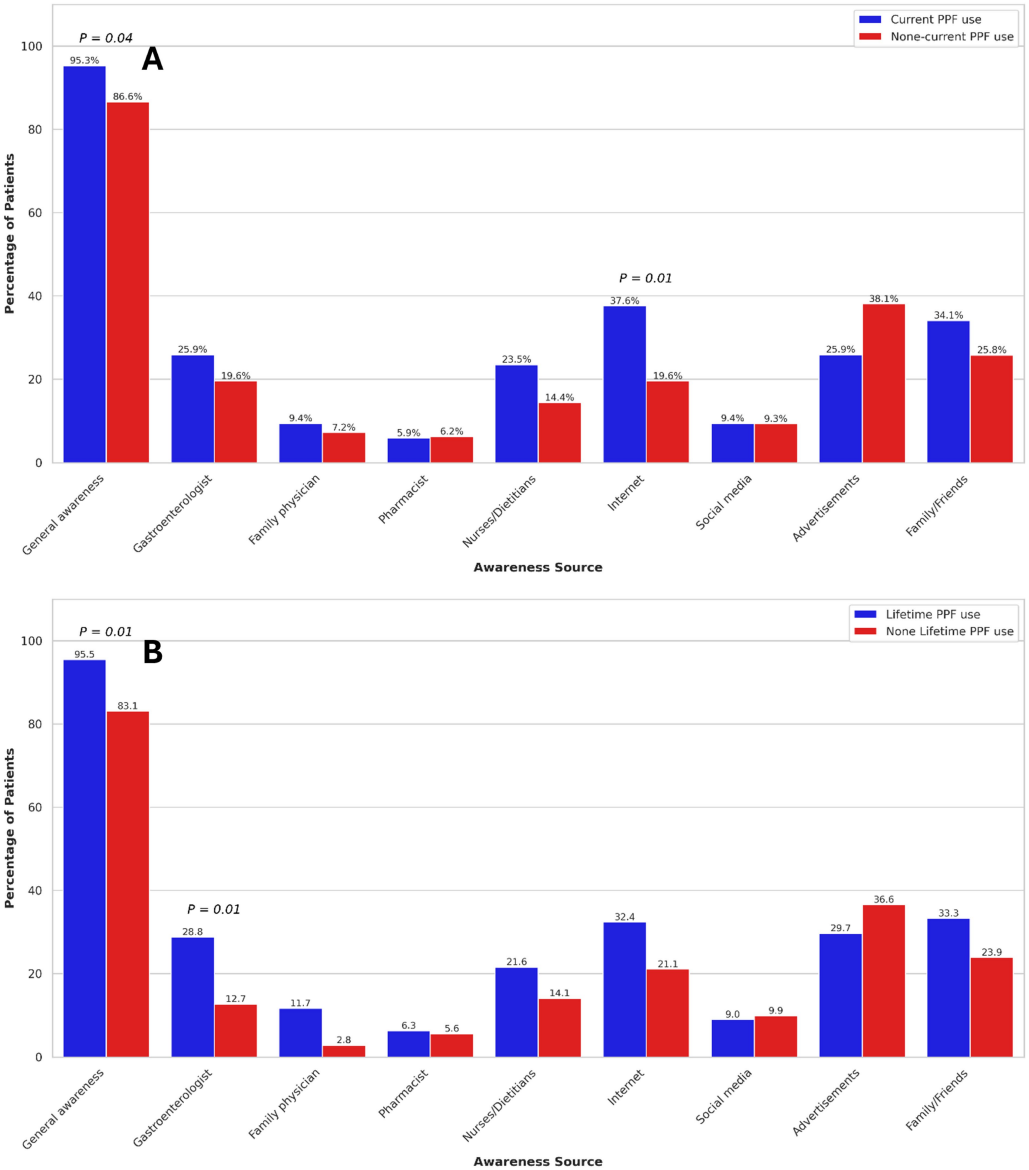
Association of current **(A)** and lifetime **(B)** probiotics, prebiotics, and fermentable dietary fibres use with sources of awareness in Crohn’s disease patients. Only *p*-values < 0.05 are shown.

The primary sources of PPF awareness among participants were advertisements (32.2%), family or friends (30.3%), the internet (28.1%), and gastroenterologists (23.6%). Among participants, 39.0% of those without a university degree and 54.2% of those with a university degree reported being aware of PPFs through healthcare professionals (*p* = 0.03), suggesting that higher educational attainment may be associated with greater awareness of these modalities. In UC patients, no significant association was found between sources of PPF awareness and current or lifetime PPF use (Figure 2). However, in CD patients, awareness through the internet was significantly associated with increased current PPF use (OR: 2.48, 95% CI: 1.27–4.83). Additionally, among CD patients, awareness through gastroenterologists (OR: 2.79, 95% CI: 1.24–6.28) was significantly associated with higher odds of lifetime PPF use (Figure 3). Among participants, 39.0% of those without a university degree and 54.2% of those with a university degree reported being aware of PPFs through healthcare professionals (*p* = 0.03), suggesting that higher educational attainment may be associated with greater awareness of these modalities.

In multivariable analysis, after adjusting for IBD subtypes and gender, sources of PPF awareness were not associated with current PPF use. However, PPF awareness through gastroenterologists (OR: 3.19, 95% CI: 1.55–6.58), family or friends (OR: 1.94, 95% CI: 1.07–3.56), family physicians (OR: 3.67, 95% CI: 1.01–13.35), and nurses or dietitians (OR: 2.16, 95% CI: 1.01–4.61) were significantly associated with increased odds of lifetime PPF use.

## Discussion

In the present study, we found a high prevalence of CIM (92.1% in the past year) and PPF (63% in lifetime) use among our cohort of IBD patients. Among the various demographic and clinical factors examined, UC diagnosis and higher educational attainment were significantly associated with increased current and lifetime consumption of PPFs, respectively. Furthermore, we identified that awareness disseminated through healthcare providers, particularly gastroenterologists, as well as through family and friends, played a substantial role in influencing the utilization of PPF products.

The use of CIM is prevalent globally, particularly among patients with chronic conditions such as UC and CD. IBD patients frequently employ various CIM modalities to manage their disease, alleviate symptoms, or enhance overall well-being (6). Motivations for CIM use often include dissatisfaction with conventional therapies or a preference for more “natural” and “safer” alternatives (21). In the present study, about 90% of participants reported using at least one form of CIM within the past year. This aligns with findings from other studies, which have demonstrated high rates of CAM use among IBD patients, exceeding 80% in some studies (21, 22). A recent survey of 230 IBD patients in British Columbia, Canada, found that 84% of participants had utilized CAM over the past year (22). Variations in CAM/CIM usage across the literature may be attributed to differences in study populations and methodological approaches, including the definition of CIM. Despite this, there appears to be a strong desire among IBD patients to explore and employ CIM modalities for symptom management.

Several demographic and clinical factors, including female gender, higher education level, long-term disease progression, and prolonged steroid use, have been suggested to influence CAM utilization among IBD patients in some studies (6). Overall, no significant associations were observed between CIM use and most of the evaluated demographic and clinical characteristics in our study. Although the association between gender and CIM use did not reach statistical significance (*p* = 0.06), there was a trend suggesting that female patients were more likely to use CIM compared to their male counterparts. This finding is consistent with results from prior studies (23–25), highlighting the role of gender as a key determinant in CIM utilization among individuals with IBD.

This study found that the lifetime and current PPF use among the participants were 63 and 51%, respectively. Furthermore, PPFs emerged as the second most commonly utilized form of CIM, following dietary supplements. This high prevalence of PPF use, particularly probiotics, aligns with findings from previous studies, which have identified probiotics as one of the most frequently used CAM modalities among IBD patients (21, 22, 24, 26–28). For example, Klemm et al. reported that approximately 55% of IBD patients at a tertiary care referral center in Vancouver, Canada, utilized probiotics, making it the most common CAM modality in their cohort (22).

Our study showed that patients with UC were nearly twice as likely to consume PPFs within the past year compared to those with CD. This finding is consistent with a recent meta-analysis (16), which suggested that combining 5-ASA with probiotics may be beneficial for inducing remission in mild-to-moderate UC, reducing the odds of recurrence in relapsing pouchitis, and trended toward reducing clinical recurrence in inactive UC decreasing clinical recurrence in inactive UC. In contrast, probiotics did not demonstrate a significant therapeutic effect in CD (16), which may explain the observed difference in PPF use between UC and CD patients in our cohort. Notably, this meta-analysis—the largest to date evaluating probiotics in IBD—rated the certainty of evidence as low for induction of clinical and endoscopic remission in UC, and very low for prevention of clinical recurrence and other outcomes. Subgroup analyses indicated that only multi-strain probiotic formulations outperformed comparators in achieving remission and preventing recurrence in UC (16). However, evidence remains limited regarding factors such as dosage, treatment duration, specific strains or combinations, and the optimal timing throughout the disease course. These findings highlight the importance of disease-specific therapeutic strategies, in line with the principles of precision medicine, in IBD management. Future research is needed to clarify existing gaps, such as optimal dosage, probiotic formulations, and patient- and disease-related factors that influence PPF effectiveness. Furthermore, although generally considered safe for most people, excessive or inappropriate use of probiotics may lead to unwanted gastrointestinal (GI) symptoms (29). It may be helpful for patients to inform their physicians about probiotic use, and for physicians to routinely ask about such products to ensure safe and coordinated care.

Among different demographic and clinical characteristics investigated in this study, higher educational attainment was the only factor significantly associated with increased lifetime PPF use among IBD patients. The relationship between education level and probiotic use in IBD remains underexplored, with existing studies yielding mixed results. While some reports found no significant correlation (17, 27), others have indicated that IBD patients with higher education levels are more likely to use probiotics (24, 30), potentially due to more frequent access to information regarding their potential benefits and their higher economic affordability for purchasing and accessing complementary products (28). Consistent with this, our finding that participants with a university degree were more likely to be aware of PPFs through healthcare professionals suggests that educational attainment and health literacy may influence patient engagement with CIM modalities. This highlights the importance for clinicians to consider patients’ educational background and information access when discussing PPF use, to support informed and coordinated care.

The source of information significantly influences PPF utilization among IBD patients, shaping their perceptions and therapeutic decisions. Patients relying on internet-based sources or advertisements may be more inclined to use these products due to extensive marketing of their purported health benefits, however, often lacking robust scientific evidence (31). In contrast, patients informed by healthcare professionals are more likely to adopt evidence-based approaches, guided by clinical indication and potential risks. In this study, while advertisements, internet, and family or friends were major sources of PPF awareness, information provided by gastroenterologists was the primary driver of lifetime PPF use. This underscores the critical role of healthcare providers, particularly gastroenterologists, in educating patients and promoting informed decision-making. Consequently, healthcare professionals must remain well-informed and vigilant regarding PPF usage, as they represent the most trusted source of information for IBD patients (27). Nonetheless, awareness through family and friends also significantly impacted PPF consumption in our study, highlighting the need to address possible misinformation from non-medical sources to mitigate the risk of inappropriate use of PPF products.

Our study is the first to comprehensively investigate the determinants of CIM and PPF use in the Canadian province of Alberta, which has the highest prevalence of IBD (968 per 100,000) in Canada (32) and one of the highest prevalences in the world. However, its limitations should be considered while interpreting the results. This study was conducted at a tertiary center, where patients often have more complex disease and are actively treated. As such, clinical characteristics—such as disease course, medication use, steroid dependence, and history of surgery—may differ from the broader IBD population in the community. These factors should be considered when extrapolating our findings, as the patterns of CIM and PPF use observed in this cohort may not fully reflect those in the general IBD population. Additionally, we did not recruit a control group, preventing comparisons of PPF use and its contributing factors between IBD patients and healthy individuals or non-IBD patients. Furthermore, due to the cross-sectional design of the study, causal relationships cannot be inferred, the potential benefits of CIM and PPFs on disease-related outcomes cannot be assessed, and changes in their use over time or with relapsing–remitting IBD symptoms cannot be evaluated. In the present study, we did not collect detailed information regarding the frequency, consistency, or duration of use, nor did we account for the consumption of foods such as yogurt that may contribute to probiotic intake. Specific product types (e.g., probiotic strains, prebiotic structures, or sources of dietary fiber), as well as dosage and frequency of use, were not assessed in this study. This limitation may affect the generalizability and reproducibility of our findings. Another limitation of this study is that we did not assess the clinical outcomes of PPF use nor evaluate potential interactions with conventional IBD medications, and therefore cannot draw conclusions about their efficacy, safety, or potential additive or adverse effects in this patient population. Future prospective studies and clinical trials are needed to address these limitations and provide further insights.

In this study, we demonstrated a high prevalence of CIM and PPF use among IBD patients with female gender being associated with increased CIM utilization. The key determinants of PPF consumption were UC diagnosis and higher educational attainment. Awareness disseminated through formal healthcare providers like gastroenterologists, as well as informal sources such as family and friends, significantly influenced PPF consumption. Knowledge about usage of PPFs by IBD patients is valuable, as some of these products may have disease-reducing potential; however, their clinical efficacy needs to be confirmed in well-designed prospective longitudinal studies and randomized clinical trials. Future research should therefore focus on evaluating the efficacy, safety, and long-term outcomes of CIM and PPF use to optimize patient-centered care.

## Data Availability

The raw data supporting the conclusions of this article will be made available by the authors, without undue reservation.
